# Clinical and radiologic outcomes of pleomorphic adenoma and adenoid cystic carcinoma of the lacrimal gland

**DOI:** 10.5935/0004-2749.20230056

**Published:** 2023

**Authors:** Kübra Serbest Ceylanoğlu, Onur Konuk

**Affiliations:** 1 Ophthalmology Clinic, Ulucanlar Eye Education and Research Hospital, Health Sciences University, Ankara, Turkey.; 2 Department of Ophthalmology, Gazi University School of Medicine, Ankara,Turkey.

**Keywords:** Lacrimal apparatus/pathology, Adenoma, pleomorphic, Carcinoma, adenoid cystic, Computed tomography, x-ray, Magnetic resonance imaging, Aparelho lacrimal/patologia, Adenoma pleomorfo, Carcinoma adenoide cístico, Tomografia computadorizada por raios x, Imagem por ressonância magnética

## Abstract

**Purpose:**

To compare the radiologic and clinical features of primary lacrimal gland pleomorphic adenoma and adenoid cystic carcinoma.

**Methods:**

This study retrospectively reviewed imaging findings and medical records of lacrimal gland pleomorphic adenoma and adenoid cystic carcinoma.

**Results:**

Eleven patients with pleomorphic adenoma and 16 patients with adenoid cystic carcinoma were evaluated. There were no statistically significant differences between groups regarding age or sex. Proptosis was the most common presenting symptom in both groups. Adenoid cystic carcinomas were more likely to present with a palpable mass, diplopia, pain, and sensory loss than pleomorphic adenomas, although the differences were not statistically significant between groups. Furthermore, there were no significant differences in terms of homogeneity and globe indentation between lacrimal gland pleomorphic adenoma and adenoid cystic carcinoma on computed tomography (CT)(all p>0.05). The rates of bone invasion, tumor calcification, and wedge sign were significantly higher in adenoid cystic carcinomas, and bone remodeling was statistically significantly higher in pleomorphic adenomas, on CT(all p<0.05). Pleomorphic adenomas were significantly more likely to show well-defined margins, lobulated contours, heterogeneous contrast enhancement, and hyperintensity on T2-weighted magnetic resonance images (all p<0.05).

**Conclusion:**

When differentiating between lacrimal gland pleomorphic adenoma and adenoid cystic carcinoma, evaluation of radiologic features along with clinical features is of great importance. Lobulated contours may be a significant distinguishing radiologic feature suggesting pleomorphic adenoma.

## Abstract (pt)

**Objetivo:**

Comparar as características radiológicas e clínicas do adenoma pleomórfico primário e do carcinoma adenoide cístico da glândula lacrimal.

**Métodos:**

Este estudo revisou retrospectivamente os achados de imagem e os prontuários médicos de casos de adenoma pleomórfico e carcinoma adenoide cístico da glândula lacrimal.

**Resultados:**

Foram avaliados 11 pacientes com adenoma pleomórfico e 16 pacientes com carcinoma adenoide cístico. Não houve diferenças estatisticamente significativas em relação à idade e sexo. Proptose foi o sintoma de apresentação mais comum em ambos os grupos. Os carcinomas adenoides císticos foram mais propensos que os adenomas pleomórficos a apresentarem massas palpáveis, diplopia, dor e perda sensorial, mas essa diferença entre os grupos não foi estatisticamente significativa. Não houve diferenças estatísticas em termos de homogeneidade e indentação do globo ocular entre os dois tipos de tumores em imagens de tomografia computadorizada (p>0,05). Também à tomografia computadorizada, a invasão óssea, a calcificação do tumor e o sinal em cunha foram mais frequentes nos carcinomas adenoides císticos, enquanto a remodelação óssea foi mais frequente nos adenomas pleomórficos, com significância estatística para todas essas manifestações (p<0,05). À ressonância magnética, os adenomas pleomórficos foram significativamente mais propensos a terem margens bem definidas, contornos lobulados, realce heterogêneo pelo contraste e hiperintensidade na ressonância magnética ponderada em T2 (p<0,05).

**Conclusão:**

Ao se diferenciar o adenoma pleomórfico e o carcinoma adenoide cístico da glândula lacrimal, é muito importante avaliar as características radiológicas juntamente com as características clínicas. Os contornos lobulados podem ser uma característica radiológica significativamente distinta em favor do adenoma pleomórfico.

## INTRODUCTION

Lacrimal gland tumors are uncommon masses that account for approximately 10% of all orbital tumors^(1)^, with the most common forms being benign and malignant tumors of lacrimal gland pleomorphic adenoma (PA) and adenoid cystic carcinoma (ACC)^(2)^. Preoperative prediction of lacrimal gland tumors - whether benign or malignant - can be helpful for surgeons and is achieved by evaluating the clinical and radiologic features of the lesion. This information strongly influences the planned surgical approach, surgical margins, and conservative management in poor surgical candidates. PAs are usua­lly treated by complete excision without prior biopsy because they have a propensity to recur and undergo malignant transformation if incompletely excised, leading to increased morbidity^(3,4)^.

The ability to differentiate PAs and ACCs based on clinical findings is limited: only a few symptoms, such as persistent pain, sensory loss, and a short duration of symptoms (<10 months) predict a diagnosis of ACC. Orbital imaging can provide crucial information for differentiating benign and malignant lacrimal gland tumors^(5)^. In addition, good knowledge of the radiologic features of lacrimal gland tumors is very important for correct diagnosis and treatment selection. In the existing literature, there are few studies examining the radiologic findings of tumors of the lacrimal gland^(6-8)^. The main purpose of this study was thus to clarify the distinctive imaging findings that may help differentiate lacrimal gland PAs and ACCs and assist in patient management.

## METHODS

We retrospectively evaluated the clinical records of patients with histopathologically-proven lacrimal gland PA and ACC who were surgically treated from 1993-2018 in a single institution. Clinical data (sex, age, and time of initial symptoms) and presenting features (proptosis, eyelid swelling, palpable mass, pain, and sensory loss) were evaluated. All patients underwent both computed tomography (CT) and magnetic resonance imaging (MRI). All participants provided written informed consent before undergoing surgery. This study was approved by the institutional review board/ethics committee of our institution (Approval number: (# 965/2018).

Detailed radiologic characteristics of the tumors were evaluated by an observer (an ophthalmologist) who was blinded to the histologic and clinical information. The anatomical extent, margins (well or poorly defined), intralesional calcification, globe indentation, bony remodeling, bone invasion, wedge sign, and contrast enhancement (homogeneous or heterogeneous) on CT were recorded. Contour (lobulated or not lobulated), T1-T2-weighted signal intensity (hyperintense, isointense, or hypointense), and contrast enhancement (homogeneous or heterogeneous) on MRI were recorded. The density on CT and signal intensity on MRI imaging were compared with extraocular muscles(extraocular muscles are isointenseon T1-T2 MRI). Statistical analysis was performed using SPSS V.22 software (IBM, USA). Fisher’s exact test was used to compare the radiologic features between tumors.

## RESULTS

**Figure 1:**
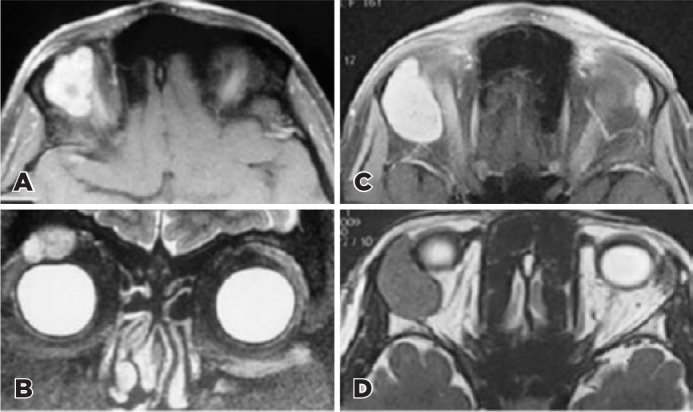
Primary lacrimal gland pleomorphic adenoma (PA; A, B) and adenoidcystic carcinoma (ACC; C, D) A: Post-contrast T1-weighted MRI shows lobulated heterogeneous contrast enhancement(PA) B: T2-weighted coronal MRI shows a hyperintense (bright) tumor(PA) C: Post-contrast T1-weighted MRI shows unlobulated homogeneous contrast enhancement (ACC) D: T2-weighted axial MRI shows a hypointense (dark) tumor (ACC) with respect toextraocular muscle.

**Figure 2:**
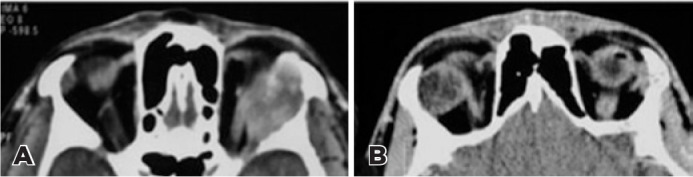
Primary lacrimal gland adenoid cystic carcinoma (ACC; A) and pleomorphic adenoma (PA; B) and (A: Calcification, invasion of bone and infiltration into the posterior orbit (wedge sign) seen left lacrimal gland adenoid cystic carcinoma on CT B: Right round, well-defined margin primary lacrimal gland pleomorphic adenoma without bone invasion.

The clinical and radiologic findings of the11 PAs and 16 ACCs were evaluated (Tables 1-2). There were no significant differences between groups in terms of age or sex. Although there was no significant difference in terms of proptosis, palpable mass, diplopia, sensory loss, or persistent pain between PAs and ACCs, these clinical symptoms were observed relatively more often in ACCs in our study. The mean duration of symptoms was significantly longer in the PA group (p=0.001);notably, duration was longer than 120 months in 5 patients with PA. Proptosis was the most common presenting symptom in both groups (90% in PA vs. 87.5% in ACC).All lacrimal gland lesions were isointense relative to extraocular muscle on CT. There were no statistically significant differences in terms of homogeneity and globe indentation between lacrimal gland PAs and ACCs on CT (p>0.05). Bone invasion, wedge sign, and calcification were observed significantly more often in ACCs, while bone remodeling was significantly more common in PAs on CT (all p<0.05). PAs were significantly more likely to show well-defined margins, lobulated contour, heterogeneous contrast enhancement, and hyperintensity on T2-weighted MRI (all p<0.05; Figures 1-2).

**Table 1:** Clinical features of pleomorphic adenoma and adenoid cystic carcinoma

Clinical features	Pleomorphic adenoma (n=11)	Adenoid cystic carcinoma (n=16)	p value
Male: Female	8:3	7:9	
Mean age (range)	34.2 (19-50)	38.4 (14-70)	0.531
Mean duration of symptoms, months (range)	69.4 (2-180)	11.8 (1-66)	0.001
Proptosis (%)	10 (90)	14 (87.5)	0.79
Swelling/palpable mass (%)	9 (81.8)	13 (81.2)	0,972
Diplopia (%)	1 (9)	4 (25)	0.618
Persistent pain (%)	0	6 (37.5)	0.054
Sensory loss (%)	0	3 (18.7)	0.248

**Table 2:** Radiological features of pleomorphic adenoma and adenoid cystic carcinoma

	Pleomorphic adenoma (n=11)	Adenoid cystic carcinoma (n=16)	p-value
**CT findings**			
Signal attenuation			
Hypodense	0	0	
Isodense	11	16	-
Hyperdense	0	0	
Contrast enhancement Homogeneity			0.072
Homogeneous	8	6	
Heterogeneous	3	10	
Globe indentation	3	3	0.617
Margin: Well defined	11	2	0.000006
Ill defined	0	14	
Anatomical extent: Wedge sign	0	14	0.000008
Tumor calcification	1	10	0.004
Bone remodeling	9	5	0.01
Invasion of bone	0	7	0.022
**MRI findings**			
T1w signal intensity			
Hyperintense	0	1	
Isointense	3	12	0.774
Hypointense	8	3	
T2w signal intensity			
Hyperintense	10	1	
Isointense	1	2	
Hypointense	0	13	0.00004
Contrast enhancement homogeneity			
Homogeneous	1	13	
Heterogeneous	10	3	0.0002
Contour			
Lobulated	10	0	
Not lobulated	1	16	0.00001

p<0.05 isconsidered statistically significant, Fisher’s exact test was used

## DISCUSSION

PA is the most common lacrimal gland epithelial tumor, accounting for 48-71% of cases, whereas the most common malignant epithelial lacrimal gland tumor ACC(12-32%)^(2,3,9)^. The prognosis for patients with PA is generally good, although it may recur or transform into malignant carcinoma ex pleomorphic adenoma after incomplete removal^(9)^. For this reason, complete resection without biopsy is recommended for PA. Data regarding the safety of preoperative incisional or fine-needle biopsy for suspected LGPA has been presented in the literature^(10)^.

In contrast, patients with ACC generally have poor long-term prognosis despite many attempts to optimize treatment regimens^(11)^.Excisional biopsy is performed in cases of suspected ACC. Fine-needle biopsy may be initially chosen in patients with advanced disease who cannot undergo total excision before radiotherapy^(12)^.Surgeons should be familiar with the radiologic findings to understand the potential diagnosis.

One aim of this study was to present similarities and differences between the clinical and radiologic findings of PA and ACC and to emphasize the radiologic findings. Lacrimal gland tumors may be found in patients of all ages, but are primarily encountered in middle-aged adults. The mean age of patients with PA in our study was 34.2 ±11.5 (range: 19-50) years, which is similar to other studies^(13,14)^. ACC has a bimodal age distribution with the majority of patients diagnosed in their 40s and a smaller peak during teenage years^(15)^.The mean age of patients with ACC in our study was 38.4 ±18.6 (range, 14-70) years, which is again similar to other studies^(3,16)^.

In our study, most PA patients were male (eight males, three females). In the literature, PAs are reported have an equal distribution between men and women or to be slightly more common in men than women with a ratio of 1.5:1^(17,18)^. The proportion of males/females with ACC in our study was 7/9. Shields et al.^(14)^ suggested that ACC is slightly more common in women, although there is no established predilection in relation to race or sex.

The longer duration of symptoms is a very important clinical indicator of PA, which correlates with the typically slow-growing nature of the tumor^(4,19)^. Symptom duration is usually 1-2 years in PA, but is reported to be as long as 20 years^(4,17-19)^. In contrast, the duration of symptoms is approximately 6 months in ACC^(19)^. In our study, there was a significant difference in symptom duration between groups in accordance with the literature (p<0.05)^(4,19)^. The rate of growth and infiltration of the surrounding tissues results in the significant difference in symptom duration between PA and ACC. In our study, in line with the literature, proptosis and palpable mass were the most common symptoms among all lacrimal gland tumors^(4,17,18)^. Although these symptoms are not distinctive, they should be thoroughly examined to evaluate the duration of the disease.

Periocular pain is an important clinical feature of lacrimal gland malignancy^(3)^. The cause of pain is reported to be the perineural spread of the tumor, especially along the ophthalmic (V1) branches of the trigeminal nerve toward the superior orbital fissure or along the zygomaticotemporal branch of the maxillary (V2) branch^(14)^. The presentation may be subtle in the superotemporal orbit. In the literature on ACC, the frequency of periocular pain has a wide range of values (35-90%)^(15,16)^. In the present study, the rate of periocular pain rate in ACC was 37.5%.

Diplopia may occur in lacrimal gland PA and ACC due to the mass effect or perineural involvement^(4,6,16,20)^. In our study, one of nine PA patients had vertical diplopia. Sensory loss was associated with diplopia in one patient with ACC. Although diplopia is not a distinguishing fea­ture of lacrimal gland benign and malignant tumors, it is more likely to be seen in ACC secondary to perineural involvement and the fast-growing nature.

In lacrimal gland malignant tumors, sensory loss may be observed in the innervation areas after invasion of the V1 or V2 sensory nerves. In the literature, trigeminal nerve function loss -typically hypoesthesia -is reported at rates of 20-33% in ACC^(16,21)^. In our study, the rate was 18% (3 of 16 cases).

All PAs showed well-defined margins on CT imaging, which is animportant sign for distinguishing benign from malignant tumors of the lacrimal gland. ACC has been reported to have poorly defined margins in the literature^(7,9,22,23)^. ACCs are also more likely to have a tail or wedge sign than PA, indicating infiltration into the posterior orbit. A triangle of tissue between the lateral rectus and lateral orbital wall or between the superior rectus and orbital roof is termed the wedge sign, and commonly appears in lacrimal gland carcinoma^(24)^. In our study, the wedge sign was observed in 14 of 16 patients with ACC. Calcification and bone invasion of masses of the lacrimal gland usually suggest malignant disease^(21)^. In our study, tumor calcification and bone invasion were observed on CT more frequently in ACC than PA (62% vs. 9% and 44% vs. 0%, respectively). In the literature, bone invasion is reported at rates of 45-82% in ACC^(22,23,25)^. Although patients with long-standing large PA may show modulation of the bony area of the lacrimal fossa without periosteal disruption (bone remodeling), this should not be considered bone invasion. Bone remodeling consists of displacement and, in most cases, thinning of bony walls. It is observed in benign neo­plasms, in some chronic inflammatory lesions, and rarely in malignant neoplasms^(26)^. Several products of malignant cells directly and indirectly cause resorption of bone and allow invading tumor cells to grow into the reabsorbed space during the bone invasion process^(27)^. On CT, bone remodeling is observed as thinning and displacement in the lacrimal fossa, whereas bone invasion is observed as destruction in the lacrimal fossa. In our study, bone remodeling was observed in 81% of patients with PA and 31% of patients with ACC. In the literature, the rates vary widely from 14 to 84% in PA^(6,7,13)^.

Although there was no significant difference in CT homogeneity between groups, heterogeneity was observed more often for ACCs. Homogeneity may vary with the cellular index of the tumors and larger tumors may be more heterogeneous due to mesenchymal stroma, cystic degeneration, necrosis, or serous/mucous collection in CT. Contrast enhancement of the lacrimal glands is better visualized on MRI than CT. Only inhomogeneous mixed-tissue tumors can be differentiated from homogeneous tumors by CT due to density differences^(28)^. As a result, CT imaging is typically more useful for assessing features like calcification, bone invasion, and margins in lacrimal gland tumors.

On MRI, PAs may appear as lobulated masses. In the presente study, lobulation of the contour was observed in 10 of 11 of PA patients; in contrast, none of the ACC patients showed lobulation of the contour in our series. Lobulated contours may thus be a specific feature for distinguishing PAs from malignant epithelial lacrimal gland tumors. Mărgăritescuet al.^(29)^ reported that PAs (prominently myxoid areas) often had incomplete capsules and tumors with characteristics that might be expected to have lobulated contours on MRI. Although there was no significant difference in T1-weighted MRI between PAs and ACCs in our study, the isointense signal density was relatively higher in ACCs. In the literature, isointensity on T1-weighted images has been observed in both epithelial and non-epithelial tumors at a similar rate^(8,25)^, and is thus not a differentiating feature for lacrimal gland tumors. In our study, PAs were more likely to present with a bright signal on T2-weighted MRI and heterogeneous nodular enhancement than ACCs. Similarly, Watanabe et al.^(6)^ and Gündüz et al.^(30)^ both reported high intensity in PA cases and a greater likelihood of contrast heterogeneous enhancement. Tsushima et al.^(31)^ reported the histologically myxoid areas of pleomorphic adenoma to be the cause of bright intensity signals on T2-weighted MRI. Young et al.^(7)^ reported that PAs showed high intensity on T2-weighted MRI and heterogeneous contrast enhancement in their series, but stated that lacrimal gland malignant epithelial tumors were more likely to have heterogeneous contrast enhancement compared with PA. However, the MRI findings of ACC, which is a subgroup of malignant epithelial tumors of the lacrimal gland, were not evaluated in that study as a separate group^(7)^. Thus,T2-weighted images may be more valuable than T1-weighted images for differential diagnosis of such patients.

The present study is subject to several limitations. This study has a retrospective design and involves a limited number of patients. There is also a need for studies that compare lacrimal gland epithelial tumors with detailed radiologic features.

In conclusion, detailed radiologic findings as well as clinical features are valuable for evaluating lacrimal gland epithelial tumors. On CT, poorly defined margins, tumor calcification, and bone invasion suggest ACC. Our findings suggest that lobulated contours may be an important distinguishing radiologic feature in favor of PA, although this is not clearly emphasized in the prior literature. Evaluating radiologic features such as lobulated contours, bright signal, and heterogeneous nodular enhancement on T2-weighted MRI together increases the probability of an accurate PA diagnosis. Ophthalmologists should thus pay attention to the aforementioned distinctive radiologic characteristics to help achieve correct diagnosis of lacrimal gland tumors.
